# The association between personal social capital and health-related quality of life among Chinese older people: A cross-sectional study

**DOI:** 10.3389/fnut.2022.995729

**Published:** 2022-12-08

**Authors:** Dongdong Jiang, Yajie Yan, Han Zhou, Quan Wang

**Affiliations:** ^1^Global Health Institute, School of Public Health, Wuhan University, Wuhan, China; ^2^School of Public Health, Zhejiang University, Hangzhou, China; ^3^Nanjing Lishui District Hospital of Traditional Chinese Medicine, Nanjing, China

**Keywords:** Chinese elderly people, personal social capital, health-related quality of life, urban-rural distribution, sociocultural aspects of health and wellbeing

## Abstract

**Background:**

Lower health-related quality of life (HRQoL) can result in adverse effects on the health of older people. This study aims to explore the relationship between personal social capital (PSC) and HRQoL among Chinese elderly people from rural-and-urban perspective.

**Materials and methods:**

4,802 samples were included from China’s health-related quality of life Survey for Older Adults 2018 (CHRQLS-OA 2018). The PSC, including bonding and bridging social capital (BOC and BRC), was measured by the Chinese version of the Personal Social Capital Scale (PSCS-16). The HRQoL was evaluated by the European Five Dimensions Questionnaire (EQ-5D-3L). Linear and Tobit regression models were conducted to examine the relationship between PSC and HRQoL.

**Results:**

The BOC and BRC of rural older people were significantly lower than those of urban older people. Pain/discomfort and anxiety/depression were the most significant health problems affecting the older samples. In the five dimensions, the proportion of rural older people with problems was higher than that of urban older people. Among rural older people, BOC was significantly related to self-rated health and EQ-5D utility index (*p* < 0.05); while BRC was insignificantly associated with self-rated health (*p* > 0.05) but related to EQ-5D utility index (*p* < 0.05). Both BOC and BRC were significantly correlated with self-rated health and EQ-5D utility index (*p* < 0.05) among urban older people.

**Conclusion:**

Our study reveals older people’s worrying PSC and HRQoL status. The relationship between PSC and HRQoL suggested that more social support and care of intimates should be encouraged to increase the PSC of older people, especially rural older people.

## Introduction

Aging has become a major global public health issue, with an estimated 1.5 billion people aged 60 and over worldwide by 2030 (1). As one low-and middle-income country with the largest population globally, the aging process in China is much faster than in many other countries worldwide in terms of growth rate and proportion (2). The Chinese population over 60 years old has been to 264.02 million, accounting for about 18.70% of the total population in 2020 (3). In contrast, the number of people over 60 years old in China is estimated to increase to 420 million by 2035 (4), indicating that China’s aging situation is becoming increasingly severe. Besides, due to the deterioration of the physical functions of the older people with ages, most of them may suffer from certain kinds of diseases, especially chronic diseases (5–7), which will directly affect their health-related quality of life (HRQoL).

According to World Health Organization (WHO), HRQoL refers to that individuals’ perception of their position in life in the context of the culture and value systems in which they live and concerning their goals, expectations, standards, and concerns (8). HRQoL reflects the multi-dimensions of health, including physiology, psychology, social function, subjective judgment, and life satisfaction (9). Developed countries first researched HRQoL and mainly focused on the population of children (10, 11), women (12, 13), and patients (14, 15). However, they pay more attention to the older people currently because aging has become one of the global public issues (16–18). Most researchers studied HRQoL of the older people on influencing factors, and have proven that demographic factors (e.g., gender, age, marital status, and living areas) (19–22), health-related behaviors (e.g., drinking, smoking) (23, 24), and chronic diseases (25) can affect the HRQoL of the older people. With the development of the economy and the change in social perception, researchers also found that socioeconomic factors such as income, educational levels, and employment were related to the HRQoL of older people (26). In addition, previous studies have also proved that social relationships (e.g., social capital) were associated with individual health (27–29).

Social capital is regarded as the sum of resources and values based on a network of personal and organizational relationships (30). It describes the characteristics of a society that can achieve common goals (31). Considering the difficulty of collecting collective social capital, most studies focus on personal social capital (PSC). PSC can be further distinguished into two dimensions: bonding social capital (BOC) and bridging social capital (BRC) (32). BOC refers to the trust and cooperation between similar members with some social demographic factors (such as age, social status, etc.), while BRC means connections between community residents whose status and power are different (30). Social capital, as a kind of actual or potential resource, many studies have proved that it plays a key role whether on a personal or collective level (33). To date, current studies have found that collective-level social capital was positive associated with HRQoL (34, 35), but limited research exists on the relationship between PSC and HRQoL of the older people. Due to the tremendous socioeconomic and health disparities between urban and rural areas in China, this study was conducted from the perspective of urban-rural differences. The hypothesis of this study is that the PSC (BOC and BRC) is related to HRQoL positively among rural and urban older people. Moreover, rare studies distinguish between BOC and BRC while elaborating on the association between PSC and HRQoL. Thus, this study aimed to explore the relationship between PSC (BOC and BRC) and HRQoL among Chinese older people. Considering the other developing countries with huge populations, such as India, Brazil, and so on, Chinese experience on the suggestions about the relationship between HRQoL and PSC among older people can offer certain reference.

## Materials and methods

### Design and participants

The date of this study was obtained from China’s Health-Related Quality of Life Survey for Older Adults 2018 (CHRQLS-OA 2018) (36). This cross-sectional survey was conducted during the Spring Festival in 2018, and intended to explore the health status of the Chinese older people aged 60 years old and above. We used convenient sampling to collect data and the survey sites including Henan province, Hubei province, Fujian province, Jiangsu province, etc. According to the study design, volunteers met the following inclusion criteria were considered as our target population: (1) individuals aged 60 years old or above, (2) individuals who voluntarily participated in our survey. But not all participants were included. Therefore, the excluded criteria were (1) individuals who could not conduct normal conversation because of aphasia, deafness, or other critical body illnesses, (2) individuals who had severe mental disorders or had been diagnosed with cognitive impairment, (3) individuals who had lost their daily living abilities. The questionnaire included participants’ sociodemographic characteristics, personal social capital, behaviors, lifestyles, mental health, HRQoL, coping styles, etc. Overall, we collected 5,638 questionnaires and 5,442 were valid after data quality control, of which 4,807 were offline samples with an effective rate of 85.26%.

Since the purpose of this study was to explore the relationship between the personal social capital of the elderly and HRQoL, respondents with missing values on personal social capital and EQ-5D were excluded. Finally, 4,802 samples of the older people aged 60 years and above were included in the study.

### Measures

#### Assessment of personal social capital

The Chinese Version of the Personal Social Capital Scale (PSCS-16) was adopted to measure PSC (37). The PSCS-16 contains 16 questions, composed of two sub-scales: BOC and BRC, both are formed from four sub-items and each sub-item contains two questions. The BOC contains (a) the perceived social network size, (b) the number of trusted social network members, (c) the number of social network members with resources (such as professional work and social influence), and (d) the number of reciprocal social network members; similarly, the BRC contains (a) perceived group size, (b) whether the group represents an individual, (c) resources owned by these groups and (d) the likelihood of getting help from the group on request (38). These response options of 16 questions were assessed using a five points Likert scale (1 = all, 2 = most, 3 = some, 4 = a few, and 5 = none). The average of two related questions’ score is the score for this sub-item, with an overall range of 8–40 points. To be consistent with the EQ-5D scores, the PSCS adopted reverse–code statistically. A higher score indicated that participants possessed more personal social capital.

The PSCS-16 has proven reliable and valid in China (19). In this study, Cronbach’s alpha of PSCS-16 total scale, BOC and BRC were 0.965, 0.932, and 0.965, and Kaiser-Meyer-Olkin (KMO) were 0.855, 0.919, and 0.953, respectively.

#### Assessment of health-related quality of life

Health-related quality of life was measured using the European Five Dimensions Questionnaire (EQ-5D-3L), which consisted of the EQ-5D descriptive system, the European Five Dimensions Questionnaire Visual Analogue Scale (EQ- VAS) and the Utility Index. The EQ-5D descriptive system measured participants’ health status in three levels of severity (no problems, moderate problems, and extreme problems) with five dimensions: Mobility (MO), Self-care (SC), Usual activities (UA), Pain/discomfort (PD), Anxiety/depression (AD) (39). The EQ-VAS score was recorded on a scale with anchor points 0 (worst health state) and 100 (best health state), which reflected their knowledge of health (40).

The EQ-5D utility index system refers to converting the combination of problems in the five dimensions of EQ-5D into a total utility score to evaluate the overall quality of life of the sample population. A higher EQ-5D utility index indicated higher levels of HRQoL (41). This study adopted the utility index system developed by Zhuo et al. (42), a model ranging from 0.1702 to 1.0000.

Previous studies have confirmed EQ-5D-3L’s reliability and validity in China (25). The Cronbach’s alpha of EQ-5D-3L was 0.786, and KMO was 0.788 in the study.

#### Basic demographic characteristics

The basic demographic information of this study included participants’ sociodemographic characteristics (gender, age, marital status, residence), socioeconomic status (annual family income per capita, educational levels, employment), number of chronic diseases and healthy behaviors (smoking, drinking, exercise, number of chronic diseases).

### Statistical analysis

Data were analyzed using Statistical Package for the Social Sciences (SPSS) version 22.0 (SPSS Inc., Chicago, IL, USA) and Stata SE 16.0, with a 95% Confidence Interval (CI) and a statistical significance level of 0.05.

Categorical variables were represented by frequencies and proportions, while metric variables were expressed as mean and standard deviation. The chi-square test was used to test whether there was a difference in sociodemographic characteristics between urban and rural areas and univariate analysis of five dimensions in EQ-5D-3L. Differences in each dimension of personal social capital between rural and urban areas, single factor analysis of EQ-VAS and EQ-5D utility index score among samples with different demographic characteristics were carried out using *T*-test and Analysis of Variance (ANOVA). The association between personal social capital and five dimensions of EQ-5D was examined by multiple linear regression, which included one initial model and four adjusted models. Linear regression and Tobit regression were, respectively used to analyse the relationship between the social capital of the older people and EQ-VAS and EQ-5D utility scores.

## Results

### General sociodemographic characteristics of respondents

As shown in Table 1, this study consisted of 4,802 older adults; all samples were divided into two groups, among whom 59.45% (*n* = 2,855) were from rural areas and 40.54% (*n* = 1,893) were from urban areas.

**TABLE 1:** Sociodemographic characteristics of respondents.

Variable	Description	Total (4,802)		Rural (2,855)		Urban (1,893)		χ^2^	*P*
		*N*	%	*N*	%	*N*	%		
Gender	Male	2,344	49.64	1,404	49.40	940	50.00	0.162	0.687
	Female	2,378	50.36	1,438	50.60	940	50.00		
Age (years)	60–64	1,033	21.83	659	23.15	374	19.83	13.106	0.001
	65–69	1,086	22.95	669	23.50	417	22.11		
	70–74	1,102	23.28	644	22.62	458	24.28		
	75–79	689	14.56	386	13.56	303	16.07		
	≥80	823	17.39	489	17.18	334	17.71		
Marital status	Married	2,995	63.29	1,707	60.02	1,288	68.22	32.834	<0.001
	Not married*	1,737	36.71	1,137	39.98	600	31.78		
Family annual income per capita (RMB)	<15,000	1,656	35.35	1,345	47.76	311	16.64	1015.495	<0.001
	15,000–30,000	1,185	25.29	854	30.33	331	17.71		
	30,000–45,000	896	19.12	400	14.20	496	26.54		
	>45,000	948	20.23	217	7.71	731	39.11		
Educational level	Illiterate	1,503	31.86	1,269	44.71	234	12.45	730.309	<0.001
	Elementary school	1,603	33.98	973	34.28	630	33.53		
	Junior high school or above	1,611	34.15	596	21.01	1,015	54.02		
Employment	Unemployed	3,297	69.81	1,760	61.80	1,537	81.97	218.360	<0.001
	Employed	1,426	30.19	1,088	38.20	338	18.03		
Number of chronic diseases	0	2,222	47.71	1,283	45.69	939	50.78	14.104	0.001
	1	1,260	27.06	770	27.42	490	26.50		
	≥2	1,175	25.23	755	26.89	420	22.72		
Smoking	Never smoke	3,216	68.15	1,834	64.58	1,382	73.55	80.573	<0.001
	Used to smoke	407	8.62	220	7.75	187	9.95		
	Smoking	1,096	23.23	786	27.68	310	16.50		
Drinking	Never drink	2,652	56.70	1,591	56.58	1,061	56.89	9.812	0.007
	Used to drink	390	8.34	208	7.40	182	9.76		
	Drinking	1,635	34.96	1,013	36.02	622	33.35		
Regular exercise	No	1,179	25.11	980	34.81	199	10.58	352.176	<0.001
	Yes	3,517	74.89	1,835	65.19	1,682	89.42		

*Not married includes divorce, separated, widowed, and never married; Sample sizes of the demographic characteristic variables may not sum to *n* = 4802 due to missing values. According to variables sorted in table, the missing data values are 69, 70, 117, 85, 85, 79, 145, 145, 83, 125, and 106, respectively.

Overall, of the participants, 49.64% were males and 50.36% were females. Nearly half of older people (44.78%) were under 70 years old, while 17.39% were over 80 years old. Most respondents (63.29%) were currently married. The annual family income per capita of less than 15,000 yuan accounted for the majority of the respondents (35.35%). Over half of the participants had received an education (68.13%), and 69.81% were reported without occupations. There were 52.29% of the samples suffered from chronic diseases, and the proportion of both non-smokers and non-drinkers was over 50% (68.15%, 56.70%, respectively). 74.89% of older people do regular exercise.

The following characteristics were found to be significant statistically differences across these two groups: age (χ^2^ = 13.106, *p* = 0.011), marital status (χ^2^ = 32.834, *p* < 0.001), family annual income per capita (χ^2^ = 1015.495, *p* < 0.001), educational level (χ2 = 730.309, *p* < 0.001), employment (χ^2^ = 218.360, *p* < 0.001), number of chronic diseases (χ^2^ = 14.104, *p* = 0.001), smoking (χ^2^ = 80.673, *p* < 0.001), drinking (χ^2^ = 9.812, *p* = 0.007), and regular exercise (χ^2^ = 352.176, *p* < 0.001).

### Scores of personal social capital of the elderly

Table 2 shows the scores of personal social capital among the participants. The respondents’ total score of personal social capital was 21.06 ± 7.33, while the score of two dimensions of personal social capital (BOC and BRC) were 11.38 ± 3.62 and 9.67 ± 4.15, respectively. The scores of the BOC and BRC among older people in rural areas were significantly lower than those in urban areas (*p* < 0.001).

**TABLE 2:** Scores of personal social capital among older people (Mean ± SD).

Variable	Total	Rural areas	Urban areas	*t*	*p*
BOC	11.38 ± 3.62	10.48 ± 3.63	12.74 ± 3.17	–22.728	<0.001
BRC	9.67 ± 4.15	8.49 ± 3.84	11.46 ± 3.96	–25.854	<0.001
PSC	21.06 ± 7.33	18.96 ± 7.02	24.20 ± 6.63	–26.085	<0.001

BOC, bonding social capital; BRC, bridging social capital; and PSC, personal social capital.

### Health status distribution on the five dimensions of European Five Dimensions Questionnaire

In this study, pain/discomfort was the most common problem among the older people: 51.52% in rural areas compared with 40.67% in urban areas (*p* < 0.001). While self-care was the least frequently reported problem: 20.91% in rural areas compared with 13.63% in urban areas (*p* < 0.001). Five dimensions of EQ-5D-3L were all statistically significant between rural and urban areas (*p* < 0.001) (Table 3).

**TABLE 3:** Health status distribution on the five dimensions of European Five Dimensions Questionnaire (EQ-5D-3L).

EQ-5D dimensions		Rural		Urban		χ^2^	*p*
		*N*	%	*N*	%		
Mobility	No problem	2,087	73.10	1,529	80.77	38.496	<0.001
	Some problems	739	25.88	344	18.17		
	Confined to bed	29	1.02	20	1.06		
Self-care	No problems	2,258	79.09	1,635	86.37	40.879	<0.001
	Some problems	543	19.02	235	12.41		
	Unable to	54	1.89	23	1.22		
Usual activities	No problem	2,050	71.80	1,494	78.92	30.837	<0.001
	Some problems	739	25.88	362	19.12		
	Unable to	66	2.31	37	1.95		
Pain/discomfort	No problems	1,384	48.48	1,123	59.32	55.794	<0.001
	Some problems	1,393	48.79	740	39.09		
	Extreme problems	78	2.73	30	1.58		
Anxiety/depression	No problem	1,844	64.59	1,535	81.09	153.491	<0.001
	Some problems	960	33.63	331	17.49		
	Extreme problems	51	1.79	27	1.43		

### Distribution of VAS scores and utility index among older people

Table 4 shows the scores of the samples’ self-rated health and utility index. The following characteristics were significantly different among the rural participants in both VAS and utility index scores: gender, age, marital status, annual family income per capita, educational attainment, employment, number of chronic diseases, drinking, and regular exercise (*p* < 0.05). Significant differences were found in VAS scores in urban samples in age, marital status, annual family income per capita, educational level, employment, number of chronic diseases, smoking, drinking, and regular exercise (*p* < 0.05). While in utility index scores only age, marital status, annual family income per capita, employment, number of chronic diseases, and regular exercise were found to be significantly different among urban samples (*p* < 0.05).

**TABLE 4:** Distribution of VAS scores and utility index among older people (Mean ± SD).

Variables		Rural areas		Urban areas	
		VAS	Utility	VAS	Utility
Gender	Male	75.14 ± 14.39	0.929 ± 0.099	80.63 ± 13.77	0.953 ± 0.088
	Female	72.97 ± 15.69	0.917 ± 0.107	77.19 ± 14.91	0.938 ± 0.103
	*T*	3.845	2.826	5.203	3.355
	*P*	<0.001	0.005	<0.001	0.001
Age (years)	60–64	75.49 ± 15.02	0.947 ± 0.081	81.13 ± 14.66	0.963 ± 0.067
	65–69	75.92 ± 14.84	0.939 ± 0.091	79.21 ± 14.60	0.949 ± 0.101
	70–74	74.51 ± 13.81	0.922 ± 0.105	78.48 ± 13.92	0.947 ± 0.101
	75–79	73.21 ± 13.89	0.915 ± 0.102	78.75 ± 13.12	0.945 ± 0.090
	≥80	69.46 ± 17.10	0873 ± 0.129	76.93 ± 15.30	0.923 ± 0.105
	*F*	16.122	43.330	3.931	7.820
	*P*	<0.001	<0.001	0.003	<0.001
Marital status	Married	76.32 ± 14.36	0.936 ± 0.099	80.79 ± 13.66	0.955 ± 0.091
	Not married	70.73 ± 15.52	0.903 ± 0.108	74.91 ± 15.25	0.925 ± 0.103
	*T*	9.869	8.109	8.046	5.908
	*P*	<0.001	<0.001	<0.001	<0.001
Family annual income per capita (RMB)	<1,500	71.51 ± 17.00	0.907 ± 0.115	73.54 ± 17.63	0.916 ± 0.132
	1,500–3,000	74.12 ± 12.74	0.924 ± 0.086	75.46 ± 15.28	0.931 ± 0.101
	3,000–4,500	78.26 ± 10.66	0.951 ± 0.094	79.78 ± 12.71	0.952 ± 0.085
	>4,500	81.43 ± 13.75	0.960 ± 0.090	82.41 ± 12.35	0.961 ± 0.071
	*F*	42.124	29.730	37.834	21.508
	*P*	<0.001	<0.001	<0.001	<0.001
Educational level	Illiterate	71.57 ± 14.11	0.909 ± 0.107	75.59 ± 14.41	0.935 ± 0.082
	Elementary school	74.87 ± 15.93	0.929 ± 0.098	79.82 ± 14.11	0.945 ± 0.109
	Junior high school and above	77.86 ± 14.73	0.942 ± 0.102	79.26 ± 14.48	0.948 ± 0.090
	*F*	38.622	22.655	7.800	1.751
	*P*	<0.001	<0.001	<0.001	0.174
Employment	Unemployed	72.74 ± 16.21	0.911 ± 0.112	78.68 ± 14.69	0.943 ± 0.100
	Employed	76.11 ± 12.82	0.943 ± 0.084	80.03 ± 13.41	0.956 ± 0.076
	*T*	–6.142	–8.795	–1.646	–2.577
	*P*	<0.001	<0.001	<0.001	0.010
Number of chronic diseases	0	77.42 ± 14.57	0.945 ± 0.093	82.53 ± 12.57	0.962 ± 0.106
	1	73.69 ± 14.34	0.924 ± 0.096	77.27 ± 14.86	0.940 ± 0.106
	≥2	68.46 ± 15.31	0.882 ± 0.117	72.78 ± 15.64	0.915 ± 0.103
	*F*	88.534	91.827	75.638	38.199
	*P*	<0.001	<0.001	<0.001	<0.001
Smoking	Never smoke	74.21 ± 14.99	0.925 ± 0.104	79.44 ± 14.35	0.947 ± 0.097
	Used to smoke	74.54 ± 15.37	0.921 ± 0.100	78.37 ± 15.04	0.951 ± 0.099
	Smoking	73.40 ± 15.27	0.922 ± 0.104	77.19 ± 14.27	0.935 ± 0.090
	*F*	0.927	1.636	3.243	2.349
	*P*	0.396	0.195	0.039	0.096
Drinking	Never drink	73.63 ± 15.12	0.921 ± 0.106	77.45 ± 15.02	0.942 ± 0.095
	Used to drink	71.23 ± 14.81	0.897 ± 0.125	81.53 ± 11.82	0.955 ± 0.061
	Drinking	75.23 ± 14.81	0.929 ± 0.095	80.81 ± 13.94	0.950 ± 0.103
	*F*	7.412	8.599	13.926	2.495
	*P*	0.001	<0.001	<0.001	0.083
Regular exercise	No	69.92 ± 13.84	0.906 ± 0.102	68.37 ± 17.64	0.871 ± 0.155
	Yes	76.18 ± 15.26	0.931 ± 0.104	80.21 ± 13.42	0.955 ± 0.082
	*T*	–11.020	–5.975	–9.164	–7.448
	*P*	<0.001	<0.001	<0.001	<0.001

### The relationship between personal social capital and European Five Dimensions Questionnaire Visual Analogue Scale

As shown in Table 5, for the rural sample, in model 1, only BOC was positively correlated with the EQ-VAS score of the older people (*B* = 0.977, 95% CI = 0.75–1.21). After adjusting for sociodemographic characteristics, socioeconomic status, number of chronic diseases, and healthy behaviors, in model 5, BOC was still positively related to the EQ-VAS score of the older people (*B* = 0.567, 95% CI = 0.32–0.81), and BRC had nothing to do with the EQ-VAS score of the elderly (*p* > 0.05).

**TABLE 5:** The relationship between personal social capital and European Five Dimensions Questionnaire Visual Analogue Scale (EQ-VAS).

		Total		Rural		Urban	
		*B* (95% CI)	S.E	*B* (95% CI)	S.E	*B* (95% CI)	S.E
Model 1	BOC	0.888 (0.714–1.062)***	0.089	0.977 (0.75–1.21)***	0.116	0.752 (0.47–1.03)***	0.144
	BRC	0.425 (0.273–0.577)***	0.078	0.118 (–0.10–0.32)	0.109	0.697 (0.47–0.92)**	0.115
Model 2	BOC	0.722 (0.545–0.899)***	0.090	0.78 (0.55–1.01)***	0.118	0.638 (0.35–0.92)***	0.146
	BRC	0.451 (0.300–0.603)***	0.077	0.192 (–0.02–0.41)	0.109	0.689 (0.46–0.92)***	0.115
Model 3	BOC	0.699 (0.517–0.882)***	0.093	0.741 (0.50–0.98)***	0.122	0.672 (0.38–0.97)***	0.151
	BRC	0.385 (0.231–0.539)***	0.079	0.266 (0.05–0.48)*	0.110	0.568 (0.34–0.80)***	0.118
Model 4	BOC	0.734 (0.557–0.911)***	0.090	0.791 (0.56–1.02)***	0.119	0.705 (0.43–0.98)***	0.142
	BRC	0.252 (0.099–0.405)**	0.078	0.042 (–0.17–0.25)	0.110	0.414 (0.19–0.64)***	0.116
Model 5	BOC	0.568 (0.383–0.753)***	0.094	0.567 (0.32–0.81)***	0.124	0.614 (0.32–0.91)***	0.151
	BRC	0.262 (0.106–0.418)**	0.079	0.209 (–0.01–0.43)	0.112	0.349 (0.12–0.58)**	0.119

*p < 0.05, **p < 0.01, ***p < 0.001; S.E, standard error. For the urban and rural samples: Model 1: the crude model of BOC and BRC, *R* = 0.315, *R*^2^ = 0.099; Model 2: adjusted for sociodemographic characteristics (gender, age, marital status), *R* = 0.350, *R*^2^ = 0.122; Model 3: adjusted for socioeconomic status (family income per capita, educational level, employment), *R* = 0.372, *R*^2^ = 0.139; Model 4: adjusted for the number of chronic diseases and healthy behaviors (number of chronic diseases, smoking, drinking, regular exercise), *R* = 0.409, *R*^2^ = 0.167; Model 5: adjusted for sociodemographic characteristics, socioeconomic status, number of chronic diseases, and healthy behaviors, *R* = 0.460, *R*^2^ = 0.212.

For the urban sample, in model 1, both the BOC (*B* = 0.752, 95% CI = 0.47–1.03) and the BRC (*B* = 0.697, 95% CI = 0.47–0.92) were related to the EQ-VAS score of the elderly positively. After adjusting for sociodemographic characteristics, socioeconomic status, number of chronic diseases, and healthy behaviors, in model 5, both the BOC (*B* = 0.614, 95% CI = 0.32–0.91) and the BRC (*B* = 0.349, 95% CI = 0.12–0.58) were still positively correlated with the EQ-VAS score of the participants.

### The relationship between personal social capital and EQ-5D utility index

As shown in Table 6, for the rural sample: in Model 1, the BOC was positively correlated with the utility score of the older people (β = 0.0111, 95% CI = 0.0089–0.0134), while the BRC was negatively correlated with the utility score of the older people (β = –0.0039, 95% CI = –0.0062–0.0017). After adjusting for sociodemographic characteristics, socioeconomic status, number of chronic diseases, and healthy behaviors, in model 5, the BOC was positively correlated with the EQ-5D utility score of the older people (β = 0.0065, 95% CI = 0.0041–0.0090), while the BRC was negatively correlated with the EQ-5D utility score of the older people (β = –0.0035, 95%-CI = –0.0057–0.0013).

**TABLE 6:** The relationship between personal social capital and utility index.

		Total			Rural areas			Urban areas		
		β	S.E	95% CI	β	S.E	95% CI	β	S.E	95% CI
Model 1	BOC	0.0009***	0.0010	0.00073–0.0111	0.0111***	0.0012	0.0089–0.0134	0.0069***	0.0018	0.0035–0.0103
	BRC	0.0015*	0.0009	0.0001–0.0003	−0.0039***	0.0011	–0.0062 to –0.0017	0.0076***	0.0014	0.0048–0.0105
Model 2	BOC	0.0068***	0.010	0.0049–0.0087	0.0086***	0.0012	0.0062–0.0109	0.0041*	0.0017	0.0006–0.0076
	BRC	0.0021*	0.0008	0.0004–0.0037	−0.0027*	0.0011	–0.0049 to –0.0006	0.0074***	0.0014	0.0045–0.0102
Model 3	BOC	0.0067***	0.0010	0.0047–0.0086	0.0082***	0.0012	0.0058 to –0.0106	0.0054**	0.0018	0.0018–0.0090
	BRC	0.0015*	0.0009	0.0002–0.0032	−0.0023*	0.0011	–0.0045 to –0.0001	0.0066***	0.0014	0.0038–0.0095
Model 4	BOC	0.0081***	0.0010	0.0061–0.0099	0.0098***	0.0012	0.0074–0.0122	0.0064***	0.0017	0.0030–0.0098
	BRC	−0.0011*	0.0008	–0.0028 to –0.005	−0.0056***	0.0011	–0.0078 to –0.0033	0.0040**	0.0014	0.0012–0.0068
Model 5	BOC	0.0051***	0.0010	0.0031–0.0071	0.0065***	0.0012	0.0041–0.0090	0.0043*	0.0017	0.0007–0.0078
	BRC	−0.0008*	0.0009	–0.0024 to –0.0009	−0.0035**	0.0011	–0.0057 to –0.0013	0.0031*	0.0014	0.0003–0.0060

*p < 0.05, **p < 0.01, ***p < 0.001; S.E, standard error. For the urban and rural samples: Model 1: the crude model of BOC and BRC, Prob > Chi^2^; Model 2: adjusted for sociodemographic characteristics (gender, age, marital status), Prob > Chi^2^; Model 3: adjusted for socioeconomic status (family income per capita, educational level, employment), Prob > Chi^2^; Model 4: adjusted for the number of chronic diseases and healthy behaviors (number of chronic diseases, smoking, drinking, regular exercise), Prob > Chi^2^; Model 5: adjusted for sociodemographic characteristics, socioeconomic status, number of chronic diseases, and healthy behaviors, Prob > Chi^2^.

For the urban sample: in Model 1, both the BOC (β = 0.0069, 95% CI = 0.0035–0.0103) and the BRC (β = 0.0076, 95% CI = 0.0048–0.0105) were positively correlated with the utility score of the older people. After adjusting for sociodemographic characteristics, socioeconomic status, number of chronic diseases, and healthy behaviors, in model 5, both the BOC (β = 0.0043, 95% CI = 0.0007–0.0078) and the BRC (β = 0.0031, 95% CI = 0.0003–0.0060) were still positively correlated with the EQ-5D utility score of the elderly.

## Discussion

To our knowledge, this is the first study that measures the relationship between personal social capital and HRQoL among Chinese older people from an urban-rural perspective. This cross-sectional study found that personal social capital was significantly associated with HRQoL among rural and urban older people. Moreover, the correlation still existed after adjusting the sociodemographic characteristics, socioeconomic status, number of chronic diseases, and healthy behaviors.

Our data showed that the total score of PSC among older people in rural areas was significantly lower than those in urban areas, which was different from a previous study (43). We speculated that the Chinese urban-rural dual structure might cause the discrepancy. In Chinese traditional culture, rural areas are more likely to be an “acquaintance society” than urban areas. On the one hand, with the development of urbanization, the young migrate to urban areas for work; on the other hand, the intimates and friends of older people start to die, resulting in the PSC of the older people in rural areas are gradually losing (44). In addition, our study also found that BRC among older people was lower than BOC both in urban and rural areas. According to the definition of BRC and BOC, it means that rural and urban areas were facing the dilemma of community or village hollowing out (45). Because there is less social participation and lacking organizations/groups that could provide community public services such as medical services and cultural services for older people (46), which may make them feel less BRC than BOC subjectively. Therefore, the government must encourage the community or village to provide services for the older people by establishing more care facilities and volunteer organizations/groups which can improve the bridge social capital of the older people and give them a sense of belonging in these social organizations or groups. Consistent with previous studies (47, 48), pain/discomfort and anxiety/depression were the most significant health problems affecting older people. In this study, the average EQ-VAS score of the older people was 76.01 ± 14.99, lower than the result of the Fifth National Health Service Survey (80.91 ± 13.7) (49), indicating that the older people were not optimistic about their self-rated health. The average utility index score was 0.9323 ± 0.1016, lower than the fifth National Health Service Survey (0.985 ± 0.056) (39), indicating the urgency of further HRQoL improvement among older adults. In addition, our study found that the utility index score of the rural sample was lower than urban samples, which calls for more attention to the HRQoL of the rural older people.

Our results showed that only BOC was positively correlated with self-rated health for the rural samples. Understandably, neighborhood mutual assistance is a normal situation, even in the current rapid economic and social development, this tradition has not died out in rural areas. Rural older people were affected by traditional values, which emphasize more on the family relationship that emphasizes the family relationship more than in urban areas. At the same time, family relationships play an essential role in the health of family members. Moreover, Stafford et al. (50) found that neighborhood relationships’ cohesion is positively related to self-rated health. Therefore, older people with more BOC have a higher level of self-rated fitness. Given that most older people in rural areas were engaged in agricultural production activities and lacked social organizations or groups, their communication scope was narrow and social participation was low (51). Another reason is that the self-esteem of rural older people is high (52), leading them to be unwilling to resort to help from social organizations/groups when experiencing health issues, which may also result in the insignificant relationship between BRC and self-rated health. In our study, both BOC and BRC were associated with self-rated health among urban older people. A previous study has proven that good interpersonal relationships and more social participation can improve the health of old citizens (53). Compared with the rural older people, the urban older people had a higher socioeconomic status and more resources to cope with adversity, which could increase their mutual communication, exchange and support, got help and encouragement from others, met their needs of economic and emotional support, relieving psychological pressure, and provided indirect protection for health (54, 55).

Our study indicated that the BOC was positively correlated with the EQ-5D utility index of all older people in rural and urban areas. That might be related to Chinese Confucianism’s filial piety and family culture (56). Traditional values have a deep-rooted influence on the Chinese, especially older people. They attach more importance to their relationship with their family, relatives, and friends (57), the support and reciprocal network provided by people close to them and their living environment had a significant role in meeting their psychological and emotional needs and promoting the quality of life of the older people (58–60). Interestingly, the BRC was negatively correlated with the utility score of the rural older people, while it was positively correlated with the EQ-5D utility score of the urban older people. The BRC was generated from the weak network between the older people and the surrounding social organizations or groups. It improved the actual value of interpersonal communication among older people through individual participation in social activities (61). Compared with older people in urban areas, due to the influence of factors such as traffic, economic conditions, ideas, and consciousness, the rural older people had relatively weak connections with the outside world, rarely participated in social activities, and had a relatively simple social network, with limited help resources available. Urban older people could get help from communities and various social organizations. In addition, as more and more older people started to use smartphones and the Internet (62, 63), especially urban older people, they had more channels to contact the outside world and obtain information. Organizational participation and citizen participation can not only help the elderly to obtain a sense of belonging and self-worth and even directly promote their physical exercise, which is conducive to health promotion. Therefore, it is suggested that government should provide social assistance for older people in multi-levels and various forms; increase health education and promotion in healthy aging; and improve physical facilities, expand coverage of old-age care.

## Limitations

There are still several limitations in our study. First, this is a cross-sectional study, which can only reflect the association between PSC and the HRQoL among older Chinese people. Therefore, causality cannot be determined. Second, this study is based on self-reported questionnaires, leading to some bias due to inaccurate responses. Third, the concept and measurement of PSC are still controversial. Though there are many ways to measure social capital, each instrument has its limitations and cannot cover all areas of social capital. For future studies, all the limitations should try to avoid. Four, we did not consider the regional and economical difference, although we conducted the survey in Henan province, Hubei province, Fujian province, Jiangsu province, etc., because we used the convenient sampling, indicating the limited representation of samples.

## Conclusion

In Conclusion, our study found that (1) the PSC of the older people needs to improve further, and the PSC level of the rural older people was lower than that of the urban areas. (2) Pain/discomfort and anxiety/depression were the most significant health problems affecting older people. Older people in rural areas were more likely to have problems than older people in urban areas, and the level of health of rural older people was worse than urban older people. (3) The PSC of the older people was related to the HRQoL. The BOC was positive associated with the rural older people’s HRQoL, while the BRC was negatively associated with the rural older people’s HRQoL. BOC and the BRC were both positively correlated with the HRQoL of urban older people. Therefore, to improve the HRQoL of the older people, we should increase the BOC of the elderly in rural areas, and the BOC and BRC of the elderly in urban areas.

## Data availability statement

The original contributions presented in this study are all included in the article. Further inquiries can be directed to the first or corresponding authors. The raw data supporting the conclusions of this article will be made available by the authors, without undue reservation.

## Ethics statement

The studies involving human participants were reviewed and approved by the Institutional Review Board, School of Public Health, and Faculty of Medical Sciences, Wuhan University. Written informed consent for participation was not required for this study in accordance with the national legislation and the institutional requirements.

## Author contributions

QW and DJ contributed to the study conception and design. DJ, YY, and HZ performed the material preparation, data collection, and analysis. DJ and YY wrote the first draft of the manuscript. QW revised and edited the draft. All authors commented on previous versions of the manuscript, read, and approved the final manuscript.
